# Effects of inter-pregnancy intervals on preterm birth, low birth weight and perinatal deaths in urban South Ethiopia: a prospective cohort study

**DOI:** 10.1186/s40748-022-00138-w

**Published:** 2022-05-11

**Authors:** Belayneh Hamdela Jena, Gashaw Andargie Biks, Yigzaw Kebede Gete, Kassahun Alemu Gelaye

**Affiliations:** 1 Department of Epidemiology and Biostatistics, Institute of Public Health, College of Medicine and Health Sciences, University of Gondar, Gondar, Ethiopia; 2 Department of Health System and Policy, Institute of Public Health, College of Medicine and Health Sciences, University of Gondar, Gondar, Ethiopia; 3 Department of Public Health, College of Medicine and Health Sciences, Wachemo University, Hossana, Ethiopia

**Keywords:** Inter-pregnancy interval, Pregnant women, Cohort, Perinatal outcomes, Ethiopia

## Abstract

**Background:**

Preterm birth, low birth weight and perinatal deaths are common adverse perinatal outcomes that are linked with each other, and a public health problems contributing to neonatal mortality, especially in developing countries. Although more than half of women in Ethiopia become pregnant within a short interval after the preceding childbirth, whether the short intervals increase the risk of adverse perinatal outcomes or not is understudied. We, therefore, aimed to assess the effects of inter-pregnancy intervals (IPIs) on the adverse perinatal outcomes.

**Methods:**

A community-based prospective cohort study was conducted among 2578 pregnant women in urban South Ethiopia. Pregnant women with IPIs < 24 months (IPIs < 18 and 18–23 months) were exposed groups, and those with IPI 24–60 months were the unexposed group. A multilevel analysis (mixed-effects) was done to estimate the effect of IPIs on preterm birth and low birth weight, and a generalized linear model for a binary outcome (fixed-effect) was done for perinatal deaths, using a 95% confidence level.

**Results:**

In this study, IPI < 18 months found to increase the risk of preterm birth (Adjusted Relative Risk (ARR) = 1.35, 95% CI: 1.02, 1.78), term low birth weight (ARR = 2.20, 95% CI: 1.35, 3.58) and perinatal deaths (ARR = 3.83, 95% CI: 1.90, 7.71) than 24–60 months. The results suggest that, about 9% of preterm birth, 21% of term low birth weight and 41% of perinatal deaths in the study population were attributed to IPI < 18 months. These could be prevented with the removal of the IPI < 18 months in the study population. IPI 18–23 months has shown no effect on the three adverse perinatal outcomes.

**Conclusion:**

This study has shown that, IPI under 18 months has a higher risk of adverse perinatal outcomes than IPI 24–60 months. Due attention should still be given for spacing pregnancies.

**Supplementary Information:**

The online version contains supplementary material available at 10.1186/s40748-022-00138-w.

## Background

We defined inter-pregnancy interval (IPI) as a time elapsed from live birth to a women’s last menstrual period (LMP) or conception [1]. Duration of IPI is one of the important factors that could affect perinatal outcomes such as preterm birth, low birth weight, small for gestational age, postpartum hemorrhage, premature rupture of membranes and preeclampsia [2–5]. However, an optimal duration of IPI is still questionable, and it pays the attention of researchers globally [6, 7].

Preterm birth, low birth weight and perinatal deaths (stillbirth and early neonatal death) are common adverse perinatal outcomes that are linked with each other, and a major public health concerns, especially in low and middle-income countries [8–10]. Globally, preterm birth is the second leading cause of death for children under 5 years, next to pneumonia [10]. Babies born preterm are also more likely to have low birth weight, which makes their survival more difficult, and leads to neonatal deaths [11, 12]. Studies reported that babies born premature and with low birth weight were more likely to die during the neonatal period of life [9, 12].

Several risk factors for preterm birth, low birth weight and perinatal deaths have been identified. To mention some: maternal education below secondary schooling, maternal age, parity, multiple gestation, pregnancy-induced hypertension, antepartum hemorrhage and premature rupture of membranes were associated with preterm birth [13–15]. Maternal age, maternal education, wealth status, maternal undernutrition, anemia, birth interval and preterm birth were associated with low birth weight [16–19]. Maternal age, maternal education, preterm delivery, anemia, birth interval, previous history of early neonatal death and low birth weight were associated with perinatal deaths [9, 20–22].

Among those factors, we interested in IPI because there are feasible interventions globally to modify IPIs like modern contraceptive methods. In Ethiopia, in particular, there are health facility and community-based platforms/strategies for implementations such as health extension programs [23]. There is a high rate of fertility and more than half of pregnancies to women in Ethiopia occur within a short duration after the preceding childbirth [24]. However, whether the short IPI (< 24 months) was linked to the adverse perinatal outcomes (preterm birth, term low birth weight and perinatal deaths) or not is understudied. Evidence from multisite community-based prospective cohort studies that elucidate the relationship between IPIs and the adverse perinatal outcomes is scarce in Ethiopian context. Thus, we hypothesized that short IPIs < 24 months (IPI < 18 months and 18–24 months) might have increased the risk of adverse perinatal outcomes than IPI 24–60 months.

Therefore, we aimed to assess the effect of short IPIs on the three adverse perinatal outcomes in urban south Ethiopia. The findings will add to the existing pieces of evidences to support recommendations and to have an in-depth understanding about to what extent preventing pregnancies that occur shortly after the preceding live births contribute to preventing the public health impacts of those adverse perinatal outcomes.

## Methods

### Study design and setting

A community-based prospective cohort study design was carried out among pregnant women in five urban settings (Hossana, Shone, Gimbichu, Jajura and Homecho) in the Hadiya zone, South Ethiopia. In this study, a total of 18 kebeles (lowest administrative unit in Ethiopia) were included.

### Participants

For this study, cohorts of pregnant women were enrolled at the end of 1^st^ trimester of confirmed pregnancy (after 12 weeks of gestation) via house-to-house identification and registration every three months, for a total of nine months. An enrolment was done from July 08, 2019 to March 30, 2020 by trained midwives. During the recruitment, study participants were included in the study based on the eligibility criteria for the exposure variable (IPI). The inclusion criteria were women who: were pregnant at the time of recruitment, had a live birth during the most recent childbirth, and were able to recall the date of last childbirth. Women who had a prior live birth ≥ 60 months earlier, a recent stillbirth, a recent abortion, and those who did not show a willingness to be followed were excluded.

### Sample size

A sample size of 2424 (exposed = 1212 and non-exposed = 1212) was calculated in Epi Info StatCalc version 7.2.2.6 software using the formula for Cohort study design, assuming % of unexposed (24–59 months interval) with outcome (low birth weight) = 8.52%, % of exposed (< 24 months interval) with outcome (low birth weight) = 11.97%, RR of 1.41, ratio of unexposed to exposed 1:1, 1-alpha of 1.96 (two sided) and 1-beta of 0.842 from previous study in Tanzania [2]. However, from July 08, 2019 to March 30, 2020, a total of 2578 pregnant women were enrolled through house-to-house identification, and the enrolled pregnant women were followed until September 30, 2020. Of 2578, 1273 were exposed groups; 769 had IPI < 18 months and 504 had IPI 18–23 months. The remained 1305 were unexposed group (IPI 24–60 months). This categorization was based on World Health Organization recommendation for pregnancy spacing [1].

### Variables and definitions

#### Outcome variables

The dependent/outcome variables were preterm birth, term low birth weight and perinatal deaths.

Preterm birth is defined as a baby born alive before 37 completed weeks (28–36 weeks) of gestation.

Term low birth weight is a live born baby’s weight at term birth (≥ 37 weeks of gestational age) less than 2500 gms.

Perinatal deaths are fetal death after 28 weeks of gestation up to 7 days, which include both stillbirth and early neonatal death.

#### Exposure variable

The exposure variable was inter-pregnancy interval (a time elapsed from live birth to subsequent conception or woman’s last menstrual period) [1].

#### Confounding variables

Confounding variables were socio-demographic, and economic and reproductive variables such as maternal age, maternal education, husband education, maternal occupation, wealth status, mode of previous delivery, age at first childbirth, parity and pregnancy intention.

### Data sources

Before baseline data collection, the questionnaire was prepared from existing related literature (published articles and Ethiopia Demographic and Health Surveys) based on the study objectives [2, 3, 24]. English version was translated to Amharic version by two native speakers of Amharic language (one was public health and the other was English language and literature in professions). Then back translation to English was done by another two individuals who could speak English (again one was from public health and the other from English language and literature). The questionnaire was pre-tested on 50 pregnant women in Durame town where the actual study population is culturally related. The investigators have amended the pre-test for unclear terms and order of questions. Baseline data about sociodemographic, economic and reproductive variables including the main exposure variable (IPI) were collected at the household level during enrolment via face-to-face interviews. Ten trained midwives collected data and five public health professionals made supervisions. The data collectors at each health facility were assigned and the list of participants was given for each of them. Outcomes (preterm birth, term low birth weight and perinatal deaths) were collected during labor, and delivery and from clients’ charts before discharge was made.

### Measurements

#### Outcomes ascertainment

Preterm birth was ascertained as a baby born alive before 37 completed weeks (36 weeks plus 6 days) but after 28 weeks of gestation. Gestational age was computed by subtracting the date of delivery from the date of childbirth and expressed in weeks. Then, gestational age was categorized as < 37 weeks (1 = preterm birth), and as ≥ 37 weeks (0 = otherwise).

Term low birth weight was ascertained as a weight of a baby who born alive at term (≥ 37 weeks of pregnancy) with a birth weight of < 2500 g. The weight categorized as < 2500 g (1 = low birth weight) and as ≥ 2500 g (0 = otherwise).

Perinatal deaths were ascertained as the death of the baby after 28 weeks of gestation and within 7 days postpartum (28 weeks up to 7 days). It includes both stillbirth (fetal death from 28 weeks of gestation up to the time of delivery, with no signs of life at birth such as fetal heartbeat, breathing, and movement) and early neonatal death (death of a live birth baby within 7 days after delivery). If at least one of them, either stillbirth or early neonatal death, happens then it was categorized as perinatal deaths (1 = yes, 0 = no).

#### Exposure ascertainment

The exposure variable (IPI) was ascertained by asking women about the date of most recent childbirth and the last menstrual period. IPI was computed by subtracting the date of recent childbirth from the date of the last menstrual period (LMP). For women who had difficulty in recalling the date of LMP, Ultrasound was used to estimate gestational age. LMP was computed by subtracting the duration of gestation, and then the value of IPI was calculated [1]. To be in line with the World Health Organization recommendation, women with IPI < 24 months were categorized as an exposed group and IPI 24–60 months as an unexposed group. During the analysis, we further categorized IPI < 24 months (the exposed group) into IPI < 18 months and 18–23 months, and then compared with IPI 24–60 months (the unexposed group).

#### Confounding ascertainment

Potential confounding variables are those variables that have an association with an exposure (IPI) variable and the outcomes (preterm birth, low birth weight and perinatal deaths). These confounders were identified by prior theoretical knowledge and literature [9, 14, 16–18, 25]. An additional figure file shows this in more detail (see Additional Fig. 1). The potential confounders were ascertained as follow: reported age at interview was measured in completed years and categorized into the 5-year interval. Educational status was measured as no formal schooling, primary education (1^st^ – 8^th^ grade), secondary education (9^th^ -12^th^ grade) and higher education (> 12^th^ grade or certificate, diploma and above). The occupation was measured by asking the women the main occupation that they routinely do. Parity was measured as the number of times a woman gives birth, irrespective of the outcomes of birth (live birth or stillbirth). Age at first childbirth was measured as reported age at the time that a woman had her first child. Mode of previous delivery refers to whether a woman has given the most recent childbirth spontaneous vaginal delivery or by other routes (caesarean section and instrumental) of delivery. Pregnancy intention was measured as whether a woman has the intention to be pregnant or not at the time of conception. Wealth index was measured using household assets for urban residence, which consists of the following items: owner of the house, number of rooms, the material of the roof, material of the floor, material of the exterior wall, source of drinking water, type of latrine, type of cooking materials (1 = electricity, 0 = wood/charcoal/biogas/natural gas, etc.), source of income, and presence or absence of; cell phone, refrigerator, radio, television, stove, chair, table, watch, modern bed, bicycle, bajaj (three wheel vehicle), motorcycle, car, donkey/horse cart, and bank account. Each item was categorized into two (1 = yes and 0 = no). Latrine and water sources were categorized as an improved and unimproved facility based on the world food program and World Health Organization recommendations. Principal component analysis was done to generate the components. Finally, the ranking was done into three quintiles (low, middle and high).

### Analysis

#### Descriptive analysis

Data were entered in Epi-data version 3.1 software and exported to R version 4.0.5 software for the analysis. Before the analysis data cleaning and recoding were done for all variables. Categorization and recoding for continuous variables were done using information from related literature. Frequencies and percentages, using cross-tabulation, were calculated for categorical variables and discreet continuous variables. For missing data, a complete case analysis approach was applied.

#### Bivariable and multivariable regression analysis

A generalized linear model for binary outcomes was used to assess the association of IPI with perinatal deaths. In the multivariable model, IPI was adjusted for all possible confounding variables, and a significant association with perinatal deaths was declared using a 95% confidence level and p < 0.05. For the other perinatal outcomes (preterm birth and term low birth weight), a multilevel generalized linear model for binary outcomes was considered due to the presence of the clustering effect, as described underneath in the model specification section. Multivariable multilevel model adjustment was done for both individual and community-level confounding variables.

#### Model specification (Multilevel generalized linear model)

This study applied a multilevel analysis technique to account for the hierarchical/clustering nature of data. The clustering variable was kebele with a cluster size of 18. In this analysis, binary response variables (preterm birth and term low birth weight) were considered for multilevel modeling, independently. A two-level multilevel generalized linear model for binary outcomes was applied in which individuals (level 1) were nested within communities (level 2). The level 1 model represents the association of individual-level factors, including IPI, with the outcome variables. The level 2 model represents the influence of community-level confounding factors on the outcomes. Four models were fitted as follows:

Model-I: It is an intercept-only model or model with no covariate inserted. It is used to check the variability among the communities (cluster-to-cluster variation) and used to give information to whether there is justifiable evidence to consider a random-effect model. Model-II: It is a multivariable model adjustment, containing only individual-level factors, including IPI. Model-III: It is a multivariable model adjustment, containing only community-level factors. Model-IV: It is a final adjustment model, containing both individual and community-level potential confounding factors.

#### Parameter estimations

The effect of IPI (fixed-effect) on preterm birth and term low birth weight was expressed by using adjusted relative risk (ARR) with 95% confidence intervals and its public health impact was interpreted using attributable fraction (AF) and population attributable fraction (PAF). AF and PAF are calculated from the adjusted RR (Appendix).

The measures of variation (random-effects) were reported by using Intra-class (community) Correlation Coefficient (ICC) which explains the percentage of variation by community-level factors (level 2). Proportional Change in community Variance (PCV) was also used to express the percentage of changes in the community-level variance between the null model (model-I) and the successive models.

## Results

### Cohort profiles

**Fig. 1:**
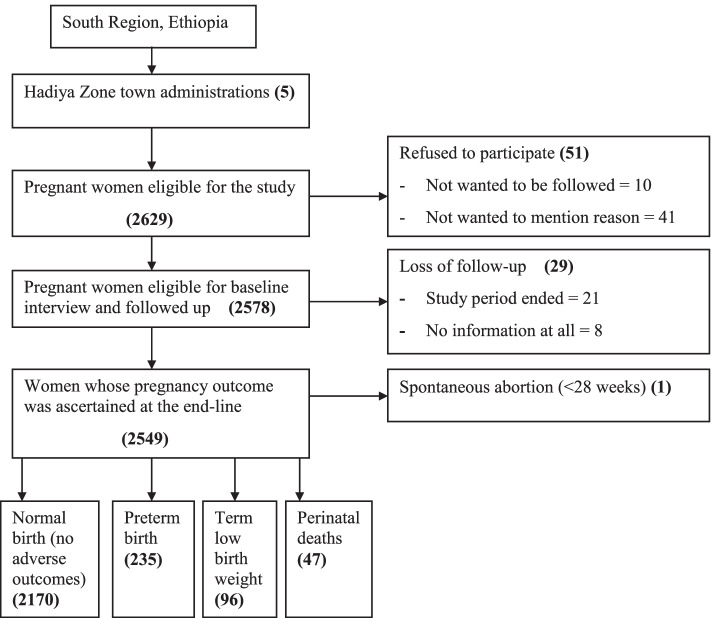
Flow-diagram of the overall study process at towns in Hadiya zone, South Ethiopia, July 2019—September 2020

A total of 2578 pregnant women were followed up until delivery. Of these, 29(1%) of them were lost of follow-up (21 due to end of the study period, 8 no information at all including via phone calling) and their pregnancy outcomes could not be ascertained. Of 29 lost of follow-up, 14 were from exposed and 15 from unexposed groups. The pregnancy outcome was ascertained for 2549 study participants. Of them, 235 had a preterm birth, 96 had term low birth weight and 47 had perinatal deaths. One woman has spontaneous abortion before 28 weeks of gestation, and she was not followed up anymore (Fig. 1).

### Individual and community-level factors of adverse perinatal outcomes

The mean age of women was 27.5 ± 3.5 years. Women with IPI under 18 months had a higher proportion of the three adverse outcomes (preterm birth, term low birth weight and perinatal deaths) than those who had IPI 24–60 months (Table 1).

**Table 1:** Individual and community-level factors of adverse perinatal outcomes in urban South Ethiopia

**Variables**	**Preterm birth (*n* = 235)**			**Term low birth weight (*n* = 96)**			**Perinatal deaths (*n* = 47)**		
	Total (*N* = 2510)	n (%)	X^2^ *P*-value	Total (*N* = 2275)	n (%)	X^2^ *P*-value	Total (*N* = 2548)	n (%)	X^2^ *P*-value
**Individual-level factors**									
**Inter-pregnancy interval in months**									
< 18	732	84 (11.5)	0.01	652	37 (5.7)	< 0.009	754	26 (3.4)	< 0.001
18–23	500	53 (10.6)		447	24 (5.4)		504	7 (1.4)	
24–60	1278	98 (7.7)		1176	35 (3.1)		1290	14 (1.1)	
**Age at interview in years**									
20–24	390	28 (7.2)	0.15	362	16 (4.4)	0.64	398	9 (2.3)	0.15
25–29	1364	127 (9.3)		1235	48 (3.9)		1386	30 (2.2)	
≥ 30	748	80 (10.7)		670	32 (4.8)		756	8 (1.1)	
**Maternal education**									
No formal education	495	71 (14.3)	< 0.001	424	24 (5.7)	0.10	501	8 (1.6)	0.89
Primary education	1055	102 (9.7)		953	45 (4.7)		1069	19 (1.8)	
Secondary education	530	41 (7.7)		489	16 (3.3)		541	12 (2.2)	
Higher education	430	21 (4.9)		409	11 (2.7)		437	8 (1.8)	
**Husband education**									
No formal education	364	56 (15.4)	< 0.001	307	25 (8.1)	0.001	370	6 (1.6)	0.47
Primary education	990	95 (9.6)		896	39 (4.4)		1000	14 (1.4)	
Secondary education	553	42 (7.6)		511	13 (2.5)		563	13 (2.3)	
Higher education	602	42 (7.0)		560	19 (3.4)		614	14 (2.3)	
**Maternal occupation**									
Housewife	1833	195 (10.6)	0.001	1637	82 (5.0)	0.011	1862	35 (1.9)	0.54
Merchant/vender/others	278	16 (5.8)		263	5 (1.9)		279	3 (1.1)	
Employed	399	24 (6.0)		375	9 (2.4)		407	9 (2.2)	
**Age at first childbirth**									
15–19	663	48 (7.2)	0.03	615	31 (5.0)	0.27	673	11 (1.6)	0.48
20–24	1511	146 (9.7)		1363	57 (4.2)		1533	27 (1.8)	
≥ 25	331	41 (12.4)		292	8 (2.7)		337	9 (2.7)	
**Parity**									
1–2	1701	139 (8.2)	0.003	1561	58 (3.7)	0.07	1729	35 (2.0)	0.34
≥ 3	804	96 (11.9)		709	38 (5.4)		814	12 (1.5)	
**Mode of previous delivery**									
Spontaneous vaginal delivery	2283	209 (9.2)	0.25	2074	85 (4.1)	0.34	2319	44 (1.9)	0.53
Cesarean-section or Instrumental	226	26 (11.5)		200	11 (5.5)		228	3 (1.3)	
**Pregnancy intention**									
Intended	1533	143 (9.3)	0.94	1383	56 (4.0)	0.59	1554	27 (1.7)	0.62
Unintended	977	92 (9.4)		888	40 (4.5)		994	20 (2.0)	
**Wealth status**									
Low	828	60 (7.2)	0.001	768	24 (3.1)	0.001	838	13 (1.6)	0.004
Medium	836	103 (12.3)		733	48 (6.5)		843	8 (0.9)	
High	827	68 (8.2)		759	23 (3.0)		848	26 (3.1)	
**Community-level factors**									
**Community maternal education**									
Low	1157	178 (15.4)	< 0.001	979	64 (6.5)	< 0.001	1038	–	–
High	1353	57 (4.2)		1296	32 (2.5)		1379	–	
**Community wealth status**									
Low	1245	153 (12.3)	< 0.001	1092	51 (4.7)	0.30	1258	–	–
High	1265	82 (6.5)		1183	45 (3.8)		1290	–	

### Effect of inter-pregnancy intervals on preterm birth and term low birth weight

#### Multivariable multilevel generalized linear model results

The null model (Model-I) showed that there was significant variability in the risk of preterm birth across the community (community variance = 0.5994, *P* < 0.05) and term low birth weight across the communities (community variance = 0.7632, *P* < 0.05). The ICC indicated that 14.41% of the variability in the risk of preterm birth and 18.83% of the variability in the risk of term low birth weight were due to community-level factors. In the final model (model-IV), the community-level variance remains significant (*P* < 0.05) even after controlling for both the individual and community-level factors. The ICC value of 9.31% in Table 2 and 9.64% in Table 3 indicate a substantial decrease in variability when community-level factors for preterm birth and term low birth weight, respectively, were included to yield more valid estimates of parameters. As shown in Table 2 and 3, the PCV value of 43.68% and 54.02% indicate that 43.68% of the variance in the risk of preterm birth and 54.02% of the variance in the risk of term low birth weight were due to the joint effects of both individual and community-level factors mentioned in the final model (model-IV) in Table 2 for preterm birth and Table 3 for term low birth weight.

**Table 2:** Multivariable multilevel generalized linear model for the effect of inter-pregnancy intervals on preterm birth

**Variable**	**Model-I (Null model)**	**Model-II** **ARR (95% CI)**	**Model-III** **ARR (95% CI)**	**Model-IV** **ARR (95% CI)**
***Fixed-effects***				
**Inter-pregnancy interval in months**				
< 18	–	1.36 (1.04, 1.80)^*^	–	1.35 (1.02, 1.78)^*^
18–23	–	1.29 (0.94, 1.77)	–	1.27 (0.92, 1.73)
24–60	–	1	–	1
***Random-effects***				
Community variance	0.5994^*^	0.5717^*^	0.3552^*^	0.3376^*^
ICC (%)	15.41	14.80	9.74	9.31
PCV (%)	–	4.62	40.74	43.68
**Model fit statistics**				
AIC	1415.8	1422.0	1413.2	1419.3
Log-likelihood	-705.9	-695.0	-702.6	-691.7

*ARR* Adjusted Relative Risk, *AIC* Akaike Information Criterion, *ICC* Intra-class Correlation Coefficient, *PCV* Proportional Change in Variance, *Model-I* Model with no covariates, *Model-II* Model with only individual-level variables adjusted, *Model-III* Model with only community-level variables adjusted, *Model-IV* Model with both individual-level and community-level variables adjusted. RR adjusted for maternal age, husband education, maternal occupation, age at first childbirth, parity, mode of previous delivery, pregnancy intention, community maternal education and community wealth status

Keys: * = *P* < 0.05, 1 = Reference category

**Table 3:** Multivariable multilevel generalized linear model for the effect of inter-pregnancy intervals on term low birth weight

**Variable**	**Model-I** **(Null model)**	**Model-II** **ARR (95% CI)**	**Model-III** **ARR (95% CI)**	**Model-IV** **ARR (95% CI)**
***Fixed-effects***				
**Inter-pregnancy interval in months**				
< 18	–	2.26 (1.39, 3.68)^**^	–	2.20 (1.35, 3.58)^**^
18–23	–	1.54 (0.91, 2.59)	–	1.48 (0.88, 2.50)
24–60	–	1	–	1
***Random-effects***				
Community variance	0.7632^*^	0.5051^*^	0.4451^*^	0.3509^*^
ICC (%)	18.83	13.31	11.92	9.64
PCV (%)	–	33.82	41.68	54.02
**Model fit statistics**				
AIC	767.7	770.7	764.9	769.6
Log-likelihood	-381.9	-369.4	-378.5	-366.8

*ARR* Adjusted Relative Risk, *AIC* Akaike Information Criterion, *ICC* Intra-class Correlation Coefficient, *PCV* Proportional Change in Variance, *Model-I* Model with no covariates, *Model-II* Model with only individual-level variables adjusted, *Model-III* Model with only community-level variables adjusted, *Model-IV* Model with both individual-level and community-level variables adjusted. RR adjusted for maternal age, husband education, maternal occupation, age at first childbirth, parity, mode of previous delivery, pregnancy intention, community maternal education and community wealth status

Keys: ** = *P* < 0.01, * = *P* < 0.05, 1 = Reference category

After adjustment for both individual and community-level confounding factors, IPI < 18 months was found to increase the risk of preterm birth. However, IPI 18–23 months was not associated with preterm birth. Accordingly, women who had a pregnancy within 18 months after a preceding live birth were 35% (ARR = 1.35, 95% CI: 1.02, 1.78) more likely to have a preterm birth as compared to women who had a pregnancy from 24–60 months (Table 2).

Among the exposure category of IPI < 18 months, about 26% (AF = 25.93%, 95%CI: 1.96, 43.82%) of preterm birth was attributed to the IPI < 18 months. In the study population, 9% (PAF = 9.26, 95% CI: 0.69, 15.64%) of preterm birth was attributed to IPI < 18 months.

After adjustment for both individual and community-level confounding factors, IPI < 18 months was found to increase the risk of term low birth weight. Women who had a pregnancy within 18 months after a preceding live birth were two times (ARR = 2.20, 95% CI: 1.35, 3.58) more likely to deliver a term low birth weight baby as compared to women who had a pregnancy from 24–60 months. IPI 18–23 months did not show significant association with term low birth weight (Table 3).

Among the exposure category of IPI < 18 months, about 54% (AF = 54.55%, 95%CI: 25.93, 72.07%) of term low birth weight was attributed to the IPI < 18 months. In the study population, 21% (PAF = 21.00, 95% CI: 9.98, 27.75%) of term low birth weight was attributed to IPI < 18 months.

#### Multicollinearity test

Collinearity diagnostic test was done for age and age at first childbirth using variance inflation factor. The maximum variance inflation factor value was 1.12, which is close to 1 or less than 10, suggesting that there was no multicollinearity problem. Thus, both variables retained in the adjusted model.

#### Model fit statistics

As indicated in Tables 2 and 3, the Akaike Information Criteria (AIC) values were subsequently decreasing and the log likelihood values were increasing for the successive models, and in the final model (model-IV) the lowest AIC and the highest log likelihood values indicate the model containing both individual and community-level factors or considering clustering effect fits the data reasonably better.

### Effect of inter-pregnancy intervals on perinatal deaths

#### Multivariable generalized linear model results

In the multivariable model, controlling for potential confounding variables, IPI under 18 months was found to increase the risk of perinatal deaths. Accordingly, women who had a pregnancy within 18 months after the preceding live birth were nearly four times (ARR = 3.83, 95% CI: 1.90, 7.71) more likely to have perinatal deaths relative to those who had a pregnancy from 24–60 months (Table 4).

**Table 4:** Multivariable generalized linear model for the effect of inter-pregnancy intervals on perinatal deaths

**Variables**	**Perinatal deaths**		**CRR (95% CI)**	**ARR (95% CI)**
	Yes (*n* = 47) n (%)	No (*n* = 2501) n (%)		
**Inter-pregnancy interval in months**				
< 18	26 (3.4)	728 (96.6)	3.18 (1.67, 6.05)^***^	3.83 (1.90, 7.71)^***^
18–23	7 (1.4)	497 (98.6)	1.28 (0.52, 3.15)	1.45 (0.58, 3.60)
24–60	14 (1.1)	1276(98.9)	1	1

*ARR* Adjusted Relative Risk, *CRR* Crude Relative Risk. RR adjusted for maternal age, maternal education, husband education, maternal occupation, age at first childbirth, parity, mode of previous delivery, pregnancy intention, and wealth status

Keys: *** = *P* < 0.001, 1 = Reference category

Among the exposure category of IPI < 18 months, about 74% (AF = 73.89%, 95%CI: 47.37, 87.03%) of perinatal deaths were attributed to the IPI < 18 months. In the study population, about 41% (PAF = 40.87, 95%CI: 26.20, 48.14) of perinatal deaths was attributed to the IPI < 18 months.

## Discussion

In this study, IPI < 18 months was found to increase the risk of preterm birth, term low birth weight and perinatal deaths as compared to IPI 24–60 months. IPI 18–23 months has shown no significant association with the three adverse perinatal outcomes.

The risk of preterm birth was higher for women with IPI under 18 months. This study suggest that about 26% of preterm births among multiparous women whose last birth was a live birth and who had an IPI < 18 months was attributed to the IPI under 18 months, which could be prevented with the removal of IPI under 18 months. In the study population (multiparous women whose last birth was a live birth and who had an IPI < 60 months), about 9% of preterm birth was attributed to the IPI < 18 months that could be prevented if IPI < 18 months did not exist. The link between IPI under 18 months and preterm birth is physiologically plausible. Shorter intervals between pregnancies might have resulted in a shorter time to recover from the abnormal conditions that happened during the preceding pregnancy and childbirth such as time to recover from the abnormal remodeling of the uterine blood vessels, which is linked to preterm premature rupture of fetal membranes and subsequent preterm births [26]. It could also be due to cervical incompetency in which the uterus failed to withstand pressure and carry the pregnancy to term as the time interval from a live birth is not adequate to recover from the preceding childbirth conditions [26]. The finding of this study is supported by previously conducted studies that reported the association of shorter IPIs with preterm birth [2, 3, 27–29]. However, the categorization of IPI varied across the studies.

In this study, IPI under 18 months was found to increase the risk of term low birth weight than the IPI 24–60 months. This study revealed that about 54% of term low birth weight was attributed to IPI under 18 months which could be prevented if the IPI under 18 months did not exist in this group (multiparous women whose last birth was a live birth and who had an IPI < 18 months). Similarly, about 21% of term low birth weight could be prevented with the removal of IPI < 18 months in the study population (multiparous women whose last birth was a live birth and who had an IPI < 60 months). The results suggest that increasing IPI to at least 18 months have advantages in increasing the weight of a baby at birth that also helps to increase survival at later ages. This association seems physiologically plausible and supports the hypothesis that short IPIs are linked to maternal depletion syndrome or pregnancy-breastfeeding overlaps that deplete maternal resources via breastfeeding for the child already born and trans-placental sharing for the fetus in the womb [26]. This, in turn, reduces the nutritional requirements of the fetus in the womb and subsequently results in low birth weight. Although the categorization of IPI varied across the studies the short IPIs found to increase the risk of low birth weight in most of the previous studies [2, 27, 29].

In this study, a higher risk of perinatal deaths was observed when IPI was under 18 months relative to IPI 24–60 months. This study reported that about 74% of perinatal deaths were attributed to IPI under 18 months that could be prevented if the IPI under 18 months did not exist in this group (multiparous women whose last birth was a live birth and who had an IPI < 18 months). About 41% of perinatal deaths could be prevented if IPI < 18 months did not exist in the study population (multiparous women whose last birth was a live birth and who had an IPI < 60 months). The exact mechanism by which short IPI results in perinatal deaths remained unclear. However, perinatal deaths might be related to the effect of IPI on preterm birth and low birth weight, as babies born preterm commonly have a low birth weight, making them unable to sustain breathing during labor and delivery and thus likely to die [9]. Babies born from mothers who have short IPI and subsequent maternal depletion are likely to have growth restriction inside the uterus, and risk of intrauterine deaths that further leads to stillbirth [26, 30]. Those who survive during the birth process are also likely to die immediately after the birth, commonly, within 7 days of delivery (early neonatal deaths) [9, 29, 31, 32]. The association of short IPIs with perinatal deaths in this study was supported by the other studies [2, 9].

This study suggests that increasing IPI to 24–60 months has health advantages and improves the health or wellbeing of the fetus/baby in the womb and immediately after delivery. It was also reported that longer intervals improve neonatal and child survival thereafter. Adequate IPI gives a resting time to women to recover from lactation stress, lost nutrients like folic acid and iron, and helps to improve maternal health status and birth outcomes in the subsequent pregnancies. IPI is a modifiable risk factor of preterm birth, low birth weight and perinatal deaths. Nowadays, feasible interventions like modern contraception are available. Modern contraceptive methods are the ideal tools that we have at hand to space pregnancies to optimal duration. Increasing IPI to optimal duration (IPI with a minimum risk of adverse perinatal outcomes) can be achieved by improving the utilization of those available modern contraceptive methods by overcoming the barriers to use such as lack of access, fear of side-effects, misconceptions, lack of decision-making power of women, lack of male involvement, etc. Family planning programs are also expected to give due emphasis to the importance of spacing pregnancies and risks such as preterm birth, low birth weight and perinatal deaths when the intervals between pregnancies are inadequate. Postnatal care might be an ideal time to counsel and initiate postpartum contraceptive utilization, including immunization visits, as these periods are a crucial time for addressing many clients, including the husband. Improving the utilization of long-acting contraceptive methods, which have a longer duration of protection than short-acting methods, may aid in achieving optimal IPI. This could be addressed through community-based development programs, such as health extension programs, in addition to health facility-based services. During the postnatal period, the risk of pregnancy might be higher due to sexual initiation since menses was not returned and couples might believe that the lactation amenorrhea method is sufficient. Women who get pregnant again after a live birth might go for an abortion, which risks bleeding and death. Thus, increasing IPI improves maternal health in addition to perinatal outcomes.

Despite the attempts made to minimize, however, this study might have limitations that the target audience needs to consider during the interpretations. Firstly, we based on women’s recall about the dates of the last childbirth and the women’s last menstrual period to calculate the IPIs, some biases related to recalling might have occurred. Secondly, although we used a multisite population-based study to reduce selection bias, still some women might have not been included in the study. Thirdly, as this study relied on community-based data collection we couldn’t collect data about previous adverse pregnancy outcomes such as history of preterm birth and low birth weight. This might have affected the estimates somehow. Fourthly, the association of IPI with perinatal deaths (rare condition) lacks precision due to small sample size. Readers need to consider this during interpretation. Despite these potential limitations, this study has strong sides: firstly, it was a multisite community-based study that provides more informative incidences of the outcomes. Secondly, it is a prospective cohort study that can elucidate the temporal relationship between exposure and the outcomes than other observational studies. Thirdly, we have also considered the effect of clustering for potential unobserved heterogeneity (cluster dependency) using a multi-level generalized linear modeling (mixed effect) for preterm birth and term low birth weight. Thus, this yields more accurate or unbiased estimates of the parameters such as variance, standard errors, and confidence intervals. Considering the aforementioned limitations, the findings of this study can be generalized for similar populations and contexts.

## Conclusion

Inter-pregnancy interval under 18 months has a higher risk of adverse perinatal outcomes than inter-pregnancy intervals 24–60 months. Short inter-pregnancy interval is a modifiable risk factor of adverse perinatal outcomes. Attention should be given for spacing pregnancies. Preventive measures like improving the utilization of modern contraceptive methods in the community could help in reducing the risk of adverse perinatal outcomes.

### Supplementary Information

**Additional file 1: Additional Figure 1.** Theoretical framework for the effect of inter-pregnancy intervals on preterm birth, term low birth weight and perinatal deaths, and potential confounding variables.

## Data Availability

The datasets used and/or analyzed during the current study are available from the corresponding author on reasonable request.

## Appendix

The AF was estimated from the ARR. AF was calculated by the formula; AF = [(RR-1)/RR]*100. PAF was calculated as; PAF = Pr(exposed/disease)*[(RR-1)/RR] = Pc*AF. Where; RR is the adjusted relative risk and Pc is the percentage of cases exposed (i.e. prevalence of exposure among the cases).

## Abbreviations

AF: Attributable Fraction
AIC: Akaike Information Criterion
ICC: Intra-class Correlation Coefficient
IPI: Inter-Pregnancy Interval
PAF: Population Attributable Fraction
PCV: Proportional Change in Variance
