# Supplementary material for: Effects of inter-pregnancy intervals on preterm birth, low birth weight and perinatal deaths in urban South Ethiopia: a prospective cohort study
Source: Matern Health Neonatol Perinatol. 2022 May 11;8:3. doi: 10.1186/s40748-022-00138-w (PMC9092840; doi:10.1186/s40748-022-00138-w)
Supplement: Supplementary file 1 — Additional file 1: Additional Figure 1. Theoretical framework for the effect of inter-pregnancy intervals on preterm birth, term low birth weight and perinatal deaths, and potential confounding variables. [file 40748_2022_138_MOESM1_ESM.docx]

Additional figure 1.

| **Exposure variable:**   - Inter-pregnancy interval   **Potential confounding variables:**   - Maternal age - Age at first child birth - Maternal education - Mode of previous delivery - Husband education - Pregnancy intention - Maternal occupation - Wealth status - Parity - History of adverse pregnancy outcomes such as history of preterm birth, low birthweight and early neonatal deaths)   **Outcome variable:**   - Preterm birth - Term low birth weight - Perinatal deaths   Additional figure 1. Theoretical frame work for the effect of inter-pregnancy interval on preterm birth, term low birth weight and perinatal deaths, and potential confounding variables. |
| --- |
